# Genomic evaluation of both purebred and crossbred performances

**DOI:** 10.1186/1297-9686-46-23

**Published:** 2014-03-25

**Authors:** Ole F Christensen, Per Madsen, Bjarne Nielsen, Guosheng Su

**Affiliations:** 1 Aarhus University, Department of Molecular Biology and Genetics, Center for Quantitative Genetics and Genomics, Blichers Allé 20, P.O. BOX 50, DK-8830 Tjele, Denmark; 2 Danish Agriculture and Food Council, Pig Research Centre, Breeding and Genetics, Axeltorv 3, DK-1609 Copenhagen V, Denmark

## Abstract

**Background:**

For a two-breed crossbreeding system, Wei and van der Werf presented a model for genetic evaluation using information from both purebred and crossbred animals. The model provides breeding values for both purebred and crossbred performances. Genomic evaluation incorporates marker genotypes into a genetic evaluation system. Among popular methods are the so-called single-step methods, in which marker genotypes are incorporated into a traditional animal model by using a combined relationship matrix that extends the marker-based relationship matrix to non-genotyped animals. However, a single-step method for genomic evaluation of both purebred and crossbred performances has not been developed yet.

**Results:**

An extension of the Wei and van der Werf model that incorporates genomic information is presented. The extension consists of four steps: (1) the Wei van der Werf model is reformulated using two partial relationship matrices for the two breeds; (2) marker-based partial relationship matrices are constructed; (3) marker-based partial relationship matrices are adjusted to be compatible to pedigree-based partial relationship matrices and (4) combined partial relationship matrices are constructed using information from both pedigree and marker genotypes. The extension of the Wei van der Werf model can be implemented using software that allows inverse covariance matrices in sparse format as input.

**Conclusions:**

A method for genomic evaluation of both purebred and crossbred performances was developed for a two-breed crossbreeding system. The method allows information from crossbred animals to be incorporated in a coherent manner for such crossbreeding systems.

## Background

Production systems based on crossbreeding are predominant in pig and chicken breeding and take advantage of the increased performance of crossbred animals compared to purebred animals. For a two-breed crossbreeding system, Wei and van der Werf (Appendix 2 in [1]) presented a model for genetic evaluation using information from both purebred and crossbred animals. The model provides estimated breeding values for purebred (mating with own breed) and crossbred (mating with the other breed) performances that are different but correlated. The model is particularly attractive since it can fit a breeding goal that includes both purebred and crossbred performances (see Jiang and Groen [2]). This model is the starting point of our paper.

Genomic selection [3] has offered a new paradigm for livestock breeding and has been successfully applied for selection within purebred populations [4-6]. Moreover, genomic selection also offers greater opportunities for incorporating information from crossbreds and selecting for crossbred performance [7-9]. Genomic selection of purebreds for crossbred performance was proposed by Ibáne~z-Escriche et al. [7] that used phenotypes on crossbreds only, and a genomic model with breed of origin specific allele substitution effects. The resulting breeding values for purebred animals were for crossbred performance. Although the study included genomic data, it was less sophisticated than the Wei and van der Werf model [1] since each animal had only one breeding value and phenotype recordings in purebreds were not used. In addition, it assumed that all relevant animals were genotyped, which would not be a very likely scenario in practice.

In cases in which not all animals are genotyped, the so-called single-step methods [10-12] provide a coherent approach for genomic evaluation. These methods incorporate marker genotypes into a traditional animal model [13] by using a combined relationship matrix that extends the marker-based relationship matrix of VanRaden [14] to non-genotyped animals, and they have been shown to perform well for genomic evaluation of dairy cattle [11,15], pigs [16,17] and chickens [18]. Misztal et al. [19] provided an extension with “unknown-parent groups” to allow for different populations, but using such an approach on data from both purebred and crossbred animals would assume equal genetic variances in the two breeds and in the crossbreds, and also that breeding values for purebred and crossbred performances are the same. A single-step method for genomic evaluation of both purebred and crossbred performances has not been developed yet.

In a genomic model, when crossbred animals are genotyped, it is natural to split the additive genetic effect of crossbreds into breed of origin specific genetic components, as in Ibáne~z-Escriche et al. [7]. Each of these components is a partial genetic effect, in the sense that only breed-specific alleles are used. This use of the terminology “partial genetic effect” is consistent with the model of Garcia-Cortes and Toro [20], in which for multibreed analysis the additive genetic value is split into several independent parts depending on their genetic origin, with the variance-covariance structure of each part being determined by a partial relationship matrix (constructed from pedigree). A partial relationship matrix is a relationship matrix that describes relationships only according to genetic origin. From this point of view, partial relationship matrices are key when constructing a single-step method for both purebred and crossbred performances. However, the Wei and van der Werf model is not formulated using partial relationship matrices, and it therefore needs to be reformulated for the purpose of incorporating genomic information.

The aim of this paper is to present an extension of the Wei van der Werf model that incorporates genomic information. The extension consists of four steps: (1) the Wei van der Werf model is reformulated using two partial relationship matrices [20] for the two breeds; (2) marker-based partial relationship matrices similar to VanRaden [14] are constructed; (3) marker-based partial relationship matrices are adjusted to be compatible to pedigree-based partial relationship matrices, similar to Christensen et al. [17] and (4) combined partial relationship matrices are constructed using information from both pedigree and marker genotypes, similar to the combined relationship matrix of Legarra et al., Aguilar et al. and Christensen and Lund [10-12]. This extension of the Wei and van der Werf model can be implemented using software that allows inverse covariance matrices in sparse format as input.

## Methods

### The Wei and van der Werf model

Here, the Wei van der Werf model (Appendix 2 in [1]) is presented. The two breeds are named  and , and it is assumed that all crossbred animals AB have known purebred parents. The number of animals in the pedigree is $n_{A}$ and $n_{B}$ for breed  and breed , respectively, and the number of crossbred animals is $n_{AB}$. The model for the phenotypes is a trivariate model

(1) $$\begin{array}{lll} & y_{A}=X_{A}\beta_{A}+Z_{A}a_{A}+e_{A},\; & \;\\ & y_{B}=X_{B}\beta_{B}+Z_{B}a_{B}+e_{B},\;\\ & y_{AB}=X_{AB}\beta_{AB}+c_{AB}+e_{AB},\; & \; \end{array}$$

where the vectors $y_{A}$, $y_{B}$ and $y_{AB}$ contain phenotypes on the breed , breed  and crossbred AB animals, respectively, and for the three breed groups ,  and AB, the vectors $X_{A}\beta_{A}$, $X_{B}\beta_{B}$ and $X_{AB}\beta_{AB}$ contain fixed effects, and $e_{A}∼N(0,\sigma_{A}^{2}I)$, $e_{B}∼N(0,\sigma_{B}^{2}I)$ and $e_{AB}∼N(0,\sigma_{AB}^{2}I)$ are the residual error vectors. The $n_{A}$-dimensional vector $a_{A}$ contains breeding values for purebred performance for breed  animals (mating within breed ), and matrix $Z_{A}$ is an incidence matrix assigning breeding values to records. Vector $a_{B}$ and matrix $Z_{B}$ are defined similarly for breed . Finally, the $n_{AB}$-dimensional vector $c_{AB}$ contains the additive genetic effects for crossbred animals, and these are related to the vectors of breeding values for purebred animals for crossbred performance (mating with the other breed) as follows

(2) $$c_{AB}=0.5({\tilde{Z}}_{AB,A}c_{A}+{\tilde{Z}}_{AB,B}c_{B})+\Phi_{AB},$$

where the matrices ${\tilde{Z}}_{AB,A}$ and ${\tilde{Z}}_{AB,B}$ assign purebred parents to crossbred offspring, $c_{A}$ is an $n_{A}$-dimensional vector containing breeding values for crossbred performance for breed  animals (mating with breed  animals), $c_{B}$ is an $n_{B}$-dimensional vector containing breeding values for crossbred performance for breed  animals (mating with breed  animals), and the vector $\Phi_{AB}$ contains the Mendelian sampling effects.

The genetic covariances are described by

$$\begin{array}{lll} & \text{Var}\left[\begin{array}{l} a_{A}\\ c_{A} \end{array}\right]=\Sigma^{\left(A\right)}⊗A_{A},\; & \;\\ & \text{Var}\left[\begin{array}{l} a_{B}\\ c_{B} \end{array}\right]=\Sigma^{\left(B\right)}⊗A_{B},\; & \;\\ & \text{Var}\left[\Phi_{AB}\right]=D_{AB},\; & \; \end{array}$$

and the three vectors are independent. The matrices $a_{A}$ and $a_{B}$ are the additive relationship matrices for breed  and breed , respectively,  denotes the Kronecker product, and

$$\Sigma^{\left(A\right)}=\left[\begin{array}{ll} \Sigma_{11}^{\left(A\right)} & \Sigma_{12}^{\left(A\right)}\\ \Sigma_{21}^{\left(A\right)} & \Sigma_{22}^{\left(A\right)} \end{array}\right]\;,$$

$$\Sigma^{\left(B\right)}=\left[\begin{array}{ll} \Sigma_{11}^{\left(B\right)} & \Sigma_{12}^{\left(B\right)}\\ \Sigma_{21}^{\left(B\right)} & \Sigma_{22}^{\left(B\right)} \end{array}\right]$$

are the 2×2 variance-covariance matrices containing the genetic variances for purebred breeding values and crossbred breeding values, and the covariance between the two, for breed  and breed , respectively. The variance-covariance matrix of the Mendelian sampling term is a diagonal matrix $D_{AB}$ with elements

(3) $$\begin{array}{lll} & {\left(D_{AB}\right)}_{\text{ii}}=\text{Var}(c_{ABi})-\frac{\text{Var}(c_{\mathrm{Af}(i)})+\text{Var}(c_{Bm(i)})}{4}\; & \;\\ & \;=(\Sigma_{22}^{\left(A\right)}+\Sigma_{22}^{\left(B\right)})/2\; & \;\\ & \;\;-(\Sigma_{22}^{\left(A\right)}{\left(A_{A}\right)}_{f(i)f(i)}+\Sigma_{22}^{\left(B\right)}{\left(A_{B}\right)}_{m(i)m(i)})/4\; & \;\\ & \;=\Sigma_{22}^{\left(A\right)}(1/2-{\left(A_{A}\right)}_{f(i)f(i)}/4)\;\\ & \;\;+\Sigma_{22}^{\left(B\right)}(1/2-{\left(A_{B}\right)}_{m(i)m(i)}/4),\; & \; \end{array}$$

where for crossbred animal *i*, *f*(*i*) denotes the breed  parent and *m*(*i*) denotes the breed  parent.

The Wei and van der Werf model is an additive genetic model in the sense that the breeding values for purebred performance, $a_{A}$, $a_{B}$, are additive genetic effects, and the breeding values for crossbred performance, $c_{A}$, $c_{B}$, in combination with the genetic effects $c_{AB}$ are also additive genetic effects. The model therefore does not contain dominance genetic effects explicitly. In practice, such an additive genetic model may also partly capture dominant gene actions and other non-additive gene actions [21]. The fact that genetic correlations between purebred and crossbred performances are different from one would be due to the presence of dominant gene actions in combination with different allele frequencies in the two breeds [22], in addition to genetic effects being different in different environments. In addition, the model captures the general level of heterosis in crossbred animals since it has a seperate fixed mean effect for crossbred animals.

Wei and van der Werf [1] made an alternative formulation of the model. The term $c_{AB}$ is not of interest for genetic evaluation when crossbred animals are not used for breeding, and Wei and van der Werf reformulated the model using $\varepsilon_{AB}=\Phi_{AB}+e_{AB}$ as the residual error term for the crossbred phenotypes and thereby expressed the model as a reduced model using only the terms $a_{A}$, $c_{A}$, $a_{B}$ and $c_{B}$ with breeding values for purebred animals. Note that due to different levels of inbreeding of parents (see formula (3)), the term $\varepsilon_{AB}$ has heterogeneous variance, and assuming a constant variance is an approximation. The reduced model can be implemented using software that handle multi-trait genetic models. For the purpose of this paper, observed marker genotypes on crossbred animals provide information on the Mendelian sampling term $\Phi_{AB}$, and the absorption of this term into the residual error term is therefore not well-suited. For this reason we do not follow the reduced model in this paper.

Finally, the special case where $\Sigma_{22}^{\left(A\right)}=\Sigma_{22}^{\left(B\right)}=\Sigma_{22}$ can be formulated as

$$\text{Var}\left[\begin{array}{c} a_{A}\\ ⋆\\ ⋆\\ ⋆\\ a_{B}\\ ⋆\\ c_{A}\\ c_{B}\\ c_{AB} \end{array}\right]=\left[\begin{array}{ccc} \Sigma_{11}^{\left(A\right)} & 0 & \Sigma_{12}^{\left(A\right)}\\ 0 & \Sigma_{11}^{\left(B\right)} & \Sigma_{12}^{\left(B\right)}\\ \Sigma_{21}^{\left(A\right)} & \Sigma_{21}^{\left(B\right)} & \Sigma_{22} \end{array}\right]⊗A,$$

where ⋆ denotes artificial random vectors such that the genetic variance-covariance matrix can be expressed using a Kronecker product, and **A** is the usual additive relationship matrix for all animals. This can therefore be implemented using a combined pedigree across all animals. We will return to this special case in the Discussion section.

### Reformulated model

Here, the Wei and van der Werf [1] model is reformulated using breed-specific partial relationship matrices, as in Garcia-Cortes and Toro [20]. Partial relationship matrices describe relationships according to genetic origin.

The starting point of the reformulation is the Mendelian sampling term for the crossbred animals in formula (2). This term can be split into breed of origin effects, $\Phi_{AB}=\Phi_{AB}^{\left(A\right)}+\Phi_{AB}^{\left(B\right)}$, where $\Phi_{AB}^{\left(A\right)}$ and $\Phi_{AB}^{\left(B\right)}$ are independent. Formula (3) can be formulated in matrix notation as $D_{AB}=\text{Var}(\Phi_{AB}^{\left(A\right)})+\text{Var}(\Phi_{AB}^{\left(B\right)})$, where $\text{Var}(\Phi_{AB}^{\left(A\right)})=\Sigma_{22}^{\left(A\right)}(0.5I_{n_{AB}}-0.25\text{diag}({\tilde{Z}}_{AB,A}A_{A}{\tilde{Z}}_{AB,A}^{T}))$ and $\text{Var}(\Phi_{AB}^{\left(B\right)})=\Sigma_{22}^{\left(B\right)}(0.5I_{n_{AB}}-0.25\text{diag}({\tilde{Z}}_{AB,B}A_{B}{\tilde{Z}}_{AB,B}^{T}))$, with $I_{n_{AB}}$ being an identity matrix of size $n_{AB}$, and $\text{diag}({\tilde{Z}}_{AB,A}A_{A}{\tilde{Z}}_{AB,A}^{T})$ and $\text{diag}({\tilde{Z}}_{AB,B}A_{B}{\tilde{Z}}_{AB,B}^{T})$ denoting diagonal matrices containing diagonal elements in matrices ${\tilde{Z}}_{AB,A}A_{A}{\tilde{Z}}_{AB,A}^{T}$ and ${\tilde{Z}}_{AB,B}A_{B}{\tilde{Z}}_{AB,B}^{T}$, respectively. In other words, we decompose the Mendelian sampling term into Mendelian sampling terms for the  and  gametes. The additive genetic effect for crossbred animals in formula (2) can then be expressed as

$$c_{AB}=c_{AB}^{\left(A\right)}+c_{AB}^{\left(B\right)},$$

where $c_{AB}^{\left(A\right)}=0.5{\tilde{Z}}_{AB,A}c_{A}+\Phi_{AB}^{\left(A\right)}$, $c_{AB}^{\left(B\right)}=0.5{\tilde{Z}}_{AB,B}c_{B}+\Phi_{AB}^{\left(B\right)}$, and $c_{AB}^{\left(A\right)}$ and $c_{AB}^{\left(B\right)}$ are independent, i.e. the genetic effects for crossbred animals is split into two breed of origin genetic effects. Thus, the model equation system (1) can be written as:

(4) $$\begin{array}{lll} & y_{A}=X_{A}\beta_{A}+Z_{A}a_{A}+e_{A},\; & \;\\ & y_{B}=X_{B}\beta_{B}+Z_{B}a_{B}+e_{B},\;\\ & y_{AB}=X_{AB}\beta_{AB}+c_{AB}^{\left(A\right)}+c_{AB}^{\left(B\right)}+e_{AB}.\; & \; \end{array}$$

Focusing on breed , the variance-covariance matrix of the genetic effects $c_{AB}^{\left(A\right)}$ becomes

$$\begin{array}{lll} & \text{Var}(c_{AB}^{\left(A\right)})=0.25\Sigma_{22}^{\left(A\right)}{\tilde{Z}}_{AB,A}A_{A}{\tilde{Z}}_{AB,A}^{T}\; & \;\\ & \;+\Sigma_{22}^{\left(A\right)}(0.5I_{n_{AB}}-0.25\text{diag}({\tilde{Z}}_{AB,A}A_{A}{\tilde{Z}}_{AB,A}^{T}))\; & \;\\ & =\Sigma_{22}^{\left(A\right)}A_{AB}^{\left(A\right)},\; & \; \end{array}$$

where $A_{AB}^{\left(A\right)}$ is a matrix with elements ${\left(A_{AB}^{\left(A\right)}\right)}_{i,i}=0.5$ and ${\left(A_{AB}^{\left(A\right)}\right)}_{i,i^{\prime }}=0.25{\left({\tilde{Z}}_{AB,A}A_{A}{\tilde{Z}}_{AB,A}^{T}\right)}_{i,i^{\prime }}$ when *i*≠*i*^′^, and the covariance matrix between $c_{AB}^{\left(A\right)}$ and $c_{A}$ becomes

$$\mathrm{Cov}(\overset{\left(A\right)}{\underset{AB}{c}},c_{A})=0.5\Sigma_{22}^{\left(A\right)}{\tilde{Z}}_{AB,A}A_{A}.$$

Therefore, the variance-covariance matrix of breed  specific genetic effects for crossbred performance equals

$$\text{Var}\left[\begin{array}{l} c_{A}\\ c_{AB}^{\left(A\right)} \end{array}\right]=\Sigma_{22}^{\left(A\right)}A^{\left(A\right)},$$

where the symmetric ($n_{A}+n_{AB}$)-dimensional matrix

() $$A^{\left(A\right)}=\left[\begin{array}{cc} A_{A} & 0.5A_{A}{\tilde{Z}}_{AB,A}^{T}\\ 0.5{\tilde{Z}}_{AB,A}A_{A} & A_{AB}^{\left(A\right)} \end{array}\right]$$

is the breed  specific partial relationship matrix in Garcia-Cortes and Toro [20] (see below).

Similarly, the variance-covariance matrix of breed  specific genetic effects for crossbred performance equals

$$\text{Var}\left[\begin{array}{c} c_{B}\\ c_{AB}^{\left(B\right)} \end{array}\right]=\Sigma_{22}^{\left(B\right)}A^{\left(B\right)},$$

where

$$A^{\left(B\right)}=\left[\begin{array}{cc} A_{B} & 0.5A_{B}{\tilde{Z}}_{AB,B}^{T}\\ 0.5{\tilde{Z}}_{AB,B}A_{B} & A_{AB}^{\left(B\right)} \end{array}\right]$$

is the breed  specific partial relationship matrix (see below).

Garcia-Cortes and Toro [20] presented a partition of the variance-covariance matrix of additive genetic values into breed-specific and breed-segregation terms, where each term is a scaled partial relationship matrix. The partial relationship matrices are constructed using recursive formulas similar to usual recursive formulas for the additive relationship matrix [23]. For the two-breed terminal crossbreeding system, the partition results in breed  and breed  specific partial relationship matrices, but no breed-segregation partial relationship matrices; we refer to Garcia-Cortes and Toro [20] for the general case. The recursive formulas for the breed  specific partial relationships are:

$$A_{\text{ii}}^{\left(A\right)}=f_{i}^{A}+A_{f(i)m(i)}^{\left(A\right)}/2,$$

$$A_{ii^{\prime }}^{\left(A\right)}=(A_{f(i)i^{\prime }}^{\left(A\right)}+A_{m(i)i^{\prime }}^{\left(A\right)})/2,$$

where *f*(*i*) and *m*(*i*) are the two parents of animal *i*, animal *i*^′^ is not a descendant of *i*, and $f_{i}^{A}$ is the breed  proportion of individual *i* (equal to 1 for purebred  animals, 0 for purebred  animals and 0.5 for crossbred animals). To insure that partial relationship matrices are invertible, Munilla-Leguizamón and Cantet [24] suggested to redefine the partial relationship matrices such that only elements that are non-null by breed origin were included, i.e. for the breed  specific partial relationships shown here, the elements related to purebred  animals are excluded. In this paper, we followed that suggestion, and it is not difficult to check that the matrix in (5) is indeed the breed  specific partial relationship matrix. Using matrix formulation, the breed  specific partial relationship matrix is $A^{\left(A\right)}=TDT^{T}$ where **D** is a diagonal matrix with elements $D_{\text{ii}}=1-(A_{f(i)f(i)}^{\left(A\right)}+A_{m(i)m(i)}^{\left(A\right)})/4$ when animal *i* is breed , and $D_{\text{ii}}=0.5-A_{f(i)f(i)}^{\left(A\right)}/4$ when animal *i* is crossbred with breed  parent *f*(*i*). For matrix **T**, the inverse matrix **T**^-1^ is a lower triangular matrix with diagonal elements equal to 1 and in the lower diagonal, the only non-zero elements are -0.5 for offspring parent elements. An example with a small pedigree is in Table 1, and the corresponding partial relationship matrices are in Tables 2 and 3.

**Table 1:** Example pedigree

**Id**	**Father**	**Mother**	**Breed group**
1	0	0	
2	0	0	
3	1	2	
4	0	0	
5	0	0	
6	4	5	
7	2	6	AB
8	3	6	AB

**Table 2:** **Breed**** specific partial relationship matrix**$A^{\left(A\right)}$** for the pedigree in Table **1

**Id**	**1**	**2**	**3**	**7**	**8**
1	1				
2	0	1			
3	1/2	1/2	1		
7	0	1/2	1/4	1/2	
8	1/4	1/4	1/2	1/8	1/2

**Table 3:** **Breed**** specific partial relationship matrix**$A^{\left(B\right)}$** for the pedigree in Table **1

**Id**	**4**	**5**	**6**	**7**	**8**
4	1				
5	0	1			
6	1/2	1/2	1		
7	1/4	1/4	1/2	1/2	
8	1/4	1/4	1/2	1/4	1/2

The reformulation of the Wei and van der Werf model is completed by introducing two artificial random vectors $A_{AB}^{\left(A\right)}$ and $a_{AB}^{\left(B\right)}$ such that genetic variance-covariance matrices can be presented using Kronecker products. For breed , the genetic covariances are described by

$$\text{Var}\left[\begin{array}{l} a_{A}\\ a_{AB}^{\left(A\right)}\\ c_{A}\\ c_{AB}^{\left(A\right)} \end{array}\right]=\Sigma^{\left(A\right)}⊗A^{\left(A\right)},$$

and similary for breed , the genetic covariances are described by

$$\text{Var}\left[\begin{array}{l} a_{B}\\ a_{AB}^{\left(B\right)}\\ c_{B}\\ c_{AB}^{\left(B\right)} \end{array}\right]=\Sigma^{\left(B\right)}⊗A^{\left(B\right)}.$$

Implementing the model requires inverses of the two partial relationship matrices. The inverse of a partial relationship matrix ${\left(A^{\left(A\right)}\right)}^{-1}$ can be expressed by the usual formula

$${\left(A^{\left(A\right)}\right)}^{-1}={\left(T^{-1}\right)}^{T}D^{-1}T^{-1},$$

and the usual methods for computing the diagonal elements of the partial relationship matrix and the inverse partial relationship matrix in sparse format [25,26] can be applied.

The model is a trivariate model with breed  and  specific genetic effects for both purebred and crossbred performances, and can be implemented using a software package for multivariate mixed models that either explicitly can construct inverses of partial relationship matrices from pedigree or alternatively can use inverse covariance matrices in sparse format as input (e.g., DMU [http://dmu.agrsci.dk], WOMBAT [http://didgeridoo.une.edu.au/km/wombat.php], ASReml [http://www.vsni.co.uk/software/asreml], blupf90 [http://nce.ads.uga.edu/wiki/doku.php]), MiX99 [http://www.mtt.fi/BGE/Software/MiX99].

Extending the model to incorporate genomic information requires the construction of two combined breed-specific partial relationship matrices expressed as inverse matrices, and for this purpose, marker-based breed-specific partial relationship matrices need to be constructed, and marker-based and pedigree-based partial relationship matrices need to be made compatible. These are the topics of the following subsections.

### Marker-based partial relationship matrix

Here, a marker-based breed  specific partial relationship matrix is constructed. The assumption here is that the marker genotypes for crossbred animals are phased such that it is known which allele originated from breed  and which allele originated from breed . The marker genotype matrix $m^{A}$ for purebred  animals has elements $m_{\text{ij}}^{A}=-$1, 0 or 1 if SNP *j* of individual *i* is 11, 12, or 22, respectively. For crossbred animals, the breed  marker allele matrix $q^{A}$ has elements $q_{\text{ij}}^{A}$ = -0.5 or 0.5 if loci *j* of individual *i* has breed  allele 1 or 2, respectively.

Constructing a marked-based breed-specific partial relationship matrix similar to the marker-based relationship matrix of VanRaden [14] is done by using the breed-specific alleles only. The marker-based breed  specific partial relationship matrix $G^{\left(A\right)}$ is divided into submatrices with indices denoting genotyped breed  and crossbred animals,

$$G^{\left(A\right)}=\left[\begin{array}{ll} G_{A,A}^{\left(A\right)} & G_{A,AB}^{\left(A\right)}\\ G_{AB,A}^{\left(A\right)} & G_{AB,AB}^{\left(A\right)} \end{array}\right],$$

which are is defined as

(5) $$\begin{array}{lll} & G_{A,A}^{\left(A\right)}=\frac{(m^{A}-(2{\bar{\rho }}^{A}-1)1^{T}){\left(m^{A}-(2{\bar{\rho }}^{A}-1)1^{T}\right)}^{T}}{s^{A}},\; & \;\\ & G_{A,AB}^{\left(A\right)}=\frac{(m^{A}-(2{\bar{\rho }}^{A}-1)1^{T}){\left(q^{A}-({\bar{\rho }}^{A}-\frac{1}{2})1^{T}\right)}^{T}}{s^{A}},\;\\ & G_{AB,AB}^{\left(A\right)}=\frac{(q^{A}-({\bar{\rho }}^{A}-\frac{1}{2})1^{T}){\left(q^{A}-({\bar{\rho }}^{A}-\frac{1}{2})1^{T}\right)}^{T}}{s^{A}},\; & \; \end{array}$$

where the vector ${\bar{\rho }}^{A}$ contains estimated breed  specific allele frequencies based on marker genotypes for purebred animals and breed  specific marker alleles for crossbred animals, and $s^{A}$ is a scaling parameter. The scaling parameter $s^{A}$ is unspecified here since we adjust the marker-based partial relationship matrix to make it compatible with the pedigree-based partial relationship matrices, similar to Christensen et al. [17] (see below).

The marker-based breed  specific partial relationship matrix $G^{\left(B\right)}$ is constructed similarly. Matrices $G^{\left(A\right)}$ and $G^{\left(B\right)}$ correspond to two different covariance structures, while matrix $G^{\left(AB\right)}$ does not exist. For crossbred animals that are genotyped, the genetic effect is the sum of two effects, with variance-covariance matrices proportional to $G_{AB,AB}^{\left(A\right)}$ and $G_{AB,AB}^{\left(B\right)}$, respectively. Since a genetic effect with a marker-based relationship matrix can be equivalently formulated as a sum of allele substitution effects, the genetic effect for crossbred animal *i* therefore equals

$$\begin{array}{lll} & c_{AB,i}=c_{AB,i}^{\left(A\right)}+c_{AB,i}^{\left(B\right)}\; & \;\\ & =\overset{p}{\underset{j=1}{\sum }}(q_{\text{ij}}^{A}-(\rho_{j}^{A}-1/2))\alpha_{j}^{A}+(q_{\text{ij}}^{B}-(\rho_{j}^{B}-1/2))\alpha_{j}^{B},\; & \; \end{array}$$

where $\alpha_{j}^{A},\alpha_{j}^{B}$ are independent breed of origin specific substitution effects for SNP *j*=1…,*p*. The model for crossbred animals is therefore as described by Ibáne~z-Escriche et al. [7].

### Compatibility of marker-based and pedigree-based partial relationship matrices

Marker-based and pedigree-based partial relationship matrices must be compatible [17,27,28]. In order to achieve this, either the marker-based partial relationship matrix or the pedigree-based partial relationship matrix must be adjusted [28]. Here, we show how to adjust the breed  specific marker-based partial relationship matrix, $G^{\left(A\right)}$, to the breed  specific pedigree-based partial relationship matrix for the subset of genotyped animals, $A_{11}^{\left(A\right)}$, similar to Christensen et al [17]. The adjustment is of the form

$$\begin{array}{lll} \;G_{a}^{\left(A\right)} & =G^{\left(A\right)}\beta +\left[\begin{array}{ll} 1_{n_{1}}{\left(1_{n_{1}}\right)}^{T} & \frac{1}{2}1_{n_{1}}{\left(1_{n_{2}}\right)}^{T}\\ \frac{1}{2}1_{n_{2}}{\left(1_{n_{1}}\right)}^{T} & \frac{1}{4}1_{n_{2}}{\left(1_{n_{2}}\right)}^{T} \end{array}\right]\alpha \; & \;\\ & =G^{\left(A\right)}\beta +K\alpha ,\; & \; \end{array}$$

with submatrices corresponding to purebred genotyped and crossbred genotyped animals, **1** denoting a vector of ones (with sub-index denoting the dimension: *n*_1_ is equal to the number of genotyped purebred animals and *n*_2_ to the number of genotyped crossbred animals); matrix **K** being implicitly defined; and *α* and *β* are parameters that need to be estimated. The form of the adjustment above is explained in Appendix Appendix A. According to Christensen et al. [17], the parameters *α* and *β* can be determined by solving a system of two equations

$${Ā}_{11}^{\left(A\right)}={\bar{G}}^{\left(A\right)}\beta +\bar{K}\alpha ,$$

$${\bar{\text{dA}}}_{11}^{\left(A\right)}={\bar{\text{dG}}}^{\left(A\right)}\beta +\bar{\text{dK}}\alpha ,$$

where ${Ā}_{11}^{\left(A\right)}$, ${\bar{G}}^{\left(A\right)}$ and $\bar{K}$ denote averages of all elements of the matrices $A_{11}^{\left(A\right)}$, $G^{\left(A\right)}$ and **K**, and ${\bar{\text{dA}}}_{11}^{\left(A\right)}$, respectively, and ${\bar{\text{dA}}}_{11}^{\left(A\right)}$, ${\bar{\text{dG}}}^{\left(A\right)}$ and $\bar{\text{dK}}$ denote averages of diagonal elements of the three matrices, respectively. Based on $\bar{K}={\left(n_{1}+n_{2}/2\right)}^{2}/{\left(n_{1}+n_{2}\right)}^{2}$ and $\bar{\text{dK}}=(n_{1}+n_{2}/4)/(n_{1}+n_{2})$, the resulting parameter estimates become

$$\beta =\frac{{Ā}_{11}^{\left(A\right)}-{\bar{\text{dA}}}_{11}^{\left(A\right)}\frac{{\left(n_{1}+n_{2}/2\right)}^{2}}{\left(n_{1}+n_{2})(n_{1}+n_{2}/4\right)}}{{\bar{G}}^{\left(A\right)}-{\bar{\text{dG}}}^{\left(A\right)}\frac{{\left(n_{1}+n_{2}/2\right)}^{2}}{\left(n_{1}+n_{2})(n_{1}+n_{2}/4\right)}},$$

$$\alpha =\frac{\left({\bar{\text{dA}}}_{11}^{\left(A\right)}-{\bar{\text{dG}}}^{\left(A\right)}\beta )(n_{1}+n_{2}\right)}{\left(n_{1}+n_{2}/4\right)}.$$

Note that parameter *β* is completely confounded with the scaling parameter $s^{A}$ in (5), and the choice of the scaling parameter is therefore irrelevant.

### Combined pedigree-based and marker-based partial relationship matrix

The combined partial relationship matrix $\left(H^{\left(A\right)}\right)$ can be constructed similar to the combined relationship matrix for purebred animals [10-12]. On the inverse scale, the elements of the matrix for non-genotyped animals do not depend on the marker genotypes. Therefore,

(6) $${\left(H^{\left(A\right)}\right)}^{-1}=\left[\begin{array}{cc} {\left(G_{\omega }^{\left(A\right)}\right)}^{-1}-{\left(A_{11}^{\left(A\right)}\right)}^{-1} & 0\\ 0 & 0 \end{array}\right]+{\left(A^{\left(A\right)}\right)}^{-1},$$

with $G_{\omega }^{\left(A\right)}=(1-\omega )G_{a}^{\left(A\right)}+\omega A_{11}^{\left(A\right)}$. Parameter *ω* is the fraction of genetic variance not captured by the marker genotypes, and in practice should be chosen to maximize accuracy and minimize bias of the resulting estimated breeding values [17].

Computation of the submatrix $A_{11}^{\left(A\right)}$ follows the Colleau algorithm [29,30], which is based on the decomposition $A^{\left(A\right)}=TDT^{T}$ shown in a previous subsection. The essential idea is to compute the *i*th column of $A_{11}^{\left(A\right)}$ by computing $A^{\left(A\right)}e_{i}$, where **e**_*i*_ is a vector with element *i* equal to 1 and all other elements equal to 0, based on Misztal et al. [30]. The algorithm consists of computing consecutively **r**=**T**^T^**e**_*i*_ by solving the sparse system (**T**^-1^)^T^**r**=**e**_*i*_ for **r**, **t**=**Dr**, and finally $A^{\left(A\right)}e_{i}=T^{T}t$ by solving the sparse system ${\left(T^{-1}\right)}^{T}(A^{\left(A\right)}e_{i})=r$ for $A^{\left(A\right)}e_{i}$.

In summary, computations for creating ${\left(H^{\left(A\right)}\right)}^{-1}$ are straightforward. First, matrices ${\left(A^{\left(A\right)}\right)}^{-1}$, $G^{\left(A\right)}$ and $A_{11}^{\left(A\right)}$ are computed, then $G^{\left(A\right)}$ is adjusted, $G_{\omega }^{\left(A\right)}=(1-\omega )G_{a}^{\left(A\right)}+\omega A_{11}^{\left(A\right)}$ is computed, and finally matrices $A_{11}^{\left(A\right)}$ and $G_{\omega }^{\left(A\right)}$ are inverted. The sparse inverse matrices ${\left(H^{\left(A\right)}\right)}^{-1}$ and ${\left(H^{\left(B\right)}\right)}^{-1}$ are used as input when implementing the extension of the Wei and van der Werf model.

## Discussion

This paper demonstrates how to incorporate marker genotypes into the Wei and van der Werf model for genetic evaluation using both purebred and crossbred information. The approach builds on using partial relationship matrices, and assumes that the marker genotypes of crossbreds can be phased such that the breed of origin of alleles is known. Many different algorithms for phasing have been developed [31,32], and it has been shown that the accuracy of phasing depends among others on size of the sample and relatedness of animals within the sample.

An alternative to using combined partial relationship matrices would be to specify one combined relationship matrix across all animals in the three breed groups ,  and AB. As mentioned in the Methods section, this is actually a special case of the model where $\Sigma_{22}^{\left(A\right)}=\Sigma_{22}^{\left(B\right)}$. With this approach, only one marker-based relationship matrix would have to be created and there would be no need to know the breed of origin of alleles. However, the adjustment of the marker-based relationship matrix to be compatible to the pedigree-based relationship matrix becomes more complicated when both breeds are considered at the same time and, as mentioned, this model is less sophisticated than the model developed in this paper.

More complicated crossbreeding systems with three breeds (mating crossbred AB animals with purebred  animals) or four breeds (mating breeds  and , mating breeds  and , and finally mating the two groups of crossbred animals AB with CD) are typically used in pig and chicken production. The three-breed crossbreeding system was studied using pig data by Ibanez et al. [33], using the Garcia-Cortes and Toro [20] decomposition of the relationship matrix, and assuming breeding values for purebred and crossbred performances were identical. However, an extension of the Wei and van der Werf model to the three-breed crossbreeding system can be formulated as follows (only the vector containing genetic effects for terminal crossbreds is shown),

$$c_{(AB)C}=c_{(AB)C}^{\left(A\right)}+c_{(AB)C}^{\left(B\right)}+c_{(AB)C}^{\left(C\right)}+c_{(AB)C}^{\left(AB\right)},$$

where the genetic terms $c_{(AB)C}^{\left(A\right)}$, $c_{(AB)C}^{\left(B\right)}$ and $c_{(AB)C}^{\left(C\right)}$ are related to the vectors containing breeding values for (AB)C crossbred performance for purebred animals, $c_{A}$, $c_{B}$ and $c_{C}$, respectively, by partial relationships. The genetic term $c_{(AB)C}^{\left(AB\right)}$ is a breed-segregation term that is independent of the other genetic terms, and has variance-covariance matrix $\Sigma_{(AB)C}^{\left(AB\right)}I_{n_{(AB)C}}/2$, where $\Sigma_{(AB)C}^{\left(AB\right)}$ is a parameter. Thus, the genetic parameter $\Sigma_{(AB)C}^{\left(AB\right)}$ and the error variance parameter $\sigma_{(AB)C}^{2}$ are not both identifiable, $c_{(AB)C}^{\left(AB\right)}$ can be incorporated into the residual error, and the three breed crossbreeding model can be formulated using three breed-specific partial relationship matrices. Extending the three-breed crossbreeding model to include observed marker genotypes is currently been investigated.

The model presented in this paper is an additive genetic model (in the sense that it considers and estimates substitution effects), but in practice it may capture both additive gene actions and partly dominant gene actions. Using purebred pig data, Su et al. [34] showed that when an additive genomic model was extended to explicitly incorporate dominance genomic effects, improved accuracies of predictions of both total genetic values and breeding values were obtained. Using simulated data from crossbred animals, Zeng et al. [9] showed that an increased response to selection was obtained with a genomic model with dominance genetic effects compared to an additive genomic model. Lo et al. [35] extended the Wei and van der Werf model to include dominance genetic effects and this model has been used in several studies on real data [36,37]. The formulation of that extension is based on extending the reduced model of Wei and van der Werf (see the Methods section) by incorporating a dominance genetic effect for the purebred phenotypes and a full-sib family effect for the crossbred phenotypes. Similar to the reduced model, this model formulation does not directly contain individual genetic effects for crossbred animals and is, therefore, not well-suited for incorporating genomic information on crossbred animals. A marker-based dominance relationship matrix was proposed by Su et al. [34], but this would need to be extended to a combined dominance relationship matrix, and further extended to a crossbreeding system. Extending the model in this paper to contain dominance genetic effects would be an interesting topic for future research.

## Conclusions

A method for genomic evaluation of both purebred and crossbred performances was developed for a two-breed crossbreeding system. The method allows information from crossbred animals to be incorporated in a coherent manner for such crossbreeding systems.

## Appendix A

In this appendix, we present the explanation behind the adjustment of the marker-based partial relationship matrix. Marker-based relationships, with allele frequencies equal to the observed ones, reflect relationships relative to the genotyped animals, whereas pedigree-based relationships are relative to the base population of the pedigree. The idea behind the adjustment of the marker-based partial relationship matrix is to translate relationships to become relative to the base population of the pedigree, instead of being relative to the given set of animals, as suggested by Powell et al. [38], and which is also the idea behind the adjustment in Christensen et al. [17].

For a given set of animals (purebred and crossbred) and a given breed, let us assume that breed-specific gametes are randomly assigned to animals (purebred animals receive two gametes each, and crossbred animals receive one gamete each), and let *α* be equal to twice the gametic relationship coefficient. The partial relationship matrix for these animals, **A**^*p*^, has entries

$$\left\{\begin{array}{ll} 1+\alpha /2 & i=i^{\prime }\;\text{purebred},\\ \alpha & i\neq i^{\prime }\;\text{purebreds},\\ \alpha /2 & i\;\text{purebred,}\;i^{\prime }\;\text{crossbred},\\ 1/2 & i=i^{\prime }\;\text{crossbreed},\\ \alpha /4 & i\neq i^{\prime }\;\text{crossbreds}, \end{array}\right)$$

and can therefore be written as

(7) $$\begin{array}{lll} A^{p} & =\left(1-\frac{\alpha }{2}\right)\left[\begin{array}{cc} I_{n_{1}} & 0\\ 0 & \frac{1}{2}I_{n_{2}} \end{array}\right]\;\\ & \;+\left[\begin{array}{ll} \alpha 1_{n_{1}}{\left(1_{n_{1}}\right)}^{T} & \frac{\alpha }{2}1_{n_{1}}{\left(1_{n_{2}}\right)}^{T}\\ \frac{\alpha }{2}1_{n_{2}}{\left(1_{n_{1}}\right)}^{T} & \frac{\alpha }{4}1_{n_{2}}{\left(1_{n_{2}}\right)}^{T} \end{array}\right],\; & \; \end{array}$$

with submatrices corresponding to purebred and crossbred animals, **1** being a vector of ones and **I** the identity matrix (with sub-indices denoting the dimension: *n*_1_ equal to number of purebred animals and *n*_2_ number of crossbred animals). The matrix

$$\left[\begin{array}{cc} I_{n_{1}} & 0\\ 0 & \frac{1}{2}I_{n_{2}} \end{array}\right]\;,$$

would be a partial relationship matrix when gametes are unrelated (*α*=0), and therefore the partial relationship matrix relative to the given set of animals. Hence, the formula (7) shows how relationships relative to the given set of animals are related to relationships relative to the base population of the pedigree. Therefore, it provides a formula to translate a marker-based relationship matrix (with allele frequencies being the observed ones) to have the same base population as the pedigree-based relationship matrix. As in Christensen et al. [17], we substitute *β* for 1-*α*/2 to incorporate the scaling parameter $s^{A}$ in (5).

## Competing interests

The authors declare that they have no competing interests.

## Authors’ contributions

OFC initiated the study, derived the methods and wrote the paper. PM, BN and GS contributed to the conception of the study and provided input to the writing of the paper. All authors have read and approved the final manuscript.

## References

[B1] Wei M van der Werf JHJ Maximizing genetic response in crossbreds using both purebred and crossbred information Anim Prod 1994 59 401 413 10.1017/S0003356100007923

[B2] Jiang X Groen AF Combined crossbred and purebred selection for reproduction traits in a broiler dam line J Anim Breed Genet 1999 116 111 125 10.1046/j.1439-0388.1999.00180.x

[B3] Meuwissen THE Hayes BJ Goddard ME Prediction of total genetic value using genome-wide dense marker maps Genetics 2001 157 1819 1829 11290733 10.1093/genetics/157.4.1819 PMC1461589

[B4] Loberg A Dürr JW Interbull survey on the use of genomic information Interbull Bull 2009 39 3 14

[B5] Brune T Stand und Perspektiven der Genomischen Selection beim Schwein Fakultät Agrarwissenschaften und Landschaftsarchitektur, Hochschule Osnabrück 2011 [Bachelorarbeit]

[B6] Fulton JE Genomic selection for poultry breeding Anim Front 2012 2 30 36 10.2527/af.2011-0028

[B7] Ibáne~z-Escriche N Fernando RL Toosi A Dekkers JCM Genomic selection of purebreds for crossbred performance Genet Sel Evol 2009 41 12 10.1186/1297-9686-41-12 19284703 PMC2730054

[B8] Kinghorn BP Hickey JM van der Werf JHJ German Society of Animal Science Reciprocal recurrent genomic selection for total genetic merit in crossbred individuals Proceedings of the 9th World Congress on Genetics Applied to Livestock Production 2010 Leipzig paper 0036. [http://www.kongressband.de/wcgalp2010/assets/pdf/0036.pdf]

[B9] Zeng J Toosi A Fernando RL Dekkers JCM Garrick DJ Genomic selection of purebred animals for crossbred performance in the presence of dominant gene action Genet Sel Evol 2013 45 11 10.1186/1297-9686-45-11 23621868 PMC3673865

[B10] Legarra A Aguilar I Misztal I A relationship matrix including full pedigree and genomic information J Dairy Sci 2009 92 4656 4663 10.3168/jds.2009-2061 19700729

[B11] Aguilar I Misztal I Johnson DL Legarra A Tsuruta S Lawlor TJ Hot topic: A unified approach to utilize phenotypic, full pedigree, and genomic information for genetic evaluations of Holstein final score J Dairy Sci 2010 93 743 752 10.3168/jds.2009-2730 20105546

[B12] Christensen OF Lund MS Genomic prediction when some animals are not genotyped Genet Sel Evol 2010 42 2 10.1186/1297-9686-42-2 20105297 PMC2834608

[B13] Henderson CR Sire evaluations and genetic trends J Anim Sci 1973 Symposium 10 41

[B14] VanRaden PM Efficient methods to compute genomic predictions J Dairy Sci 2008 91 4414 4423 10.3168/jds.2007-0980 18946147

[B15] Gao H Christensen OF Madsen P Nielsen US Zhang Y Lund MS Su G Comparison on genomic predictions using GBLUP models and two single-step blending methods with different relationship matrices in the Nordic Holstein population Genet Sel Evol 2012 44 8 10.1186/1297-9686-44-8 22455934 PMC3400441

[B16] Forni S Aguilar I Misztal I Different genomic relationship matrices for single-step analysis using phenotypic, pedigree and genomic information Genet Sel Evol 2011 43 1 10.1186/1297-9686-43-1 21208445 PMC3022661

[B17] Christensen OF Madsen P Nielsen B Ostersen T Su G Single-step methods for genomic evaluation in pigs Animal 2012 6 1565 1571 10.1017/S1751731112000742 22717310

[B18] Chen CY Misztal I Aguilar I Tsuruta S Meuwissen THE Aggrey SE Wind T Muir WM Genome-wide marker-assisted selection combining all pedigree phenotypic information with genotypic data in one-step: an example using broiler chickens J Anim Sci 2010 89 23 28 20889689 10.2527/jas.2010-3071

[B19] Misztal I Vitezica ZG Legarra A Aguilar I Swan AA Unknown-parent groups in single-step genomic evaluation J Anim Breed Genet 2013 130 252 258 10.1111/jbg.12025 23855627

[B20] García-Cortés LA Toro MA Multibreed analysis by splitting the breeding values Genet Sel Evol 2006 38 601 615 17129562 10.1186/1297-9686-38-6-601 PMC2689266

[B21] Hill WG Goddard ME Visscher PM Data and theory point to mainly additive genetic variance for complex traits PLoS Genet 2008 4 e1000008 10.1371/journal.pgen.1000008 18454194 PMC2265475

[B22] Wei M van der Werf JHJ Brascamp EW Relationship between purebred and crossbred parameters: II Genetic correlation between purebred and crossbred performance under the model with two loci J Anim Breed Genet 1991 108 262 269 10.1111/j.1439-0388.1991.tb00184.x

[B23] Henderson CR A simple method for computing the inverse of a numerator relationship matrix used in prediction of breeding values Biometrics 1976 32 69 83 10.2307/2529339

[B24] Munilla Leguizamón S Cantet RJC Equivalence of multibreed animal models and hierarchical Bayes analysis for maternally influenced traits Genet Sel Evol 2010 42 20 10.1186/1297-9686-42-20 20540758 PMC2909157

[B25] Quaas RL Computing the diagonal elements and inverse of a large numerator relationship matrix Biometrics 1976 32 949 953 10.2307/2529279

[B26] Meuwissen THE Luo Z Computing inbreeding coefficients in large populations Genet Sel Evol 1992 24 305 313 10.1186/1297-9686-24-4-305

[B27] Vitezica ZG Aguilar I Misztal I Legarra A Bias in genomic predictions for populations under selection Genet Res 2011 93 357 366 10.1017/S001667231100022X 21767459

[B28] Christensen OF Compatibility of pedigree-based and marker-based relationship matrices for single-step genetic evaluation Genet Sel Evol 2012 44 37 10.1186/1297-9686-44-37 23206367 PMC3549765

[B29] Colleau JJ An indirect approach to the extensive calculation of relationship coefficients Genet Sel Evol 2002 34 409 421 10.1186/1297-9686-34-4-409 12270102 PMC2705453

[B30] Misztal I Legarra A Aguilar I Computing procedures for genetic evaluation including phenotypic, full pedigree and genomic information J Dairy Sci 2009 92 4648 4655 10.3168/jds.2009-2064 19700728

[B31] Hickey JM Kinghorn BP Tier B Wilson JF Dunstan N van der Werf JHJ A combined long-range phasing and long haplotype imputation method to impute phase for SNP genotypes Genet Sel Evol 2011 43 12 10.1186/1297-9686-43-12 21388557 PMC3068938

[B32] Browning SR Browning BL Haplotype phasing: existing methods and new developments Nat Rev Genet 2011 12 703 714 10.1038/nrg3054 21921926 PMC3217888

[B33] Ibáne~z-Escriche N Reixach J Lleonart N Noguera JL Genetic evaluation combining purebred and crossbred data in a pig breeding scheme J Anim Sci 2011 89 3881 3889 10.2527/jas.2011-3959 21841081

[B34] Su G Christensen OF Ostersen T Henryon M Lund MS Estimating additive and non-additive genetic variances and predicting genetic merits using genome-wide dense single nucleotide polymorphism markers PLoS ONE 2012 7 e45293 10.1371/journal.pone.0045293 23028912 PMC3441703

[B35] Lo LL Fernando RL Grossman M Genetic evaluation by BLUP in two-breed terminal crossbreeding systems under dominance J Anim Sci 1997 75 2877 2884 9374299 10.2527/1997.75112877x

[B36] Lutaaya E Misztal I Mabry JW Short T Timm HH Holzbauer R Genetic parameter estimates from joint evaluation of purebreds and crossbreds in swine using the crossbred model J Anim Sci 2001 79 3002 3007 11811453 10.2527/2001.79123002x

[B37] Lutaaya E Misztal I Mabry JW Short T Timm HH Holzbauer R Joint evaluation of purebreds and crossbreds in swine J Anim Sci 2002 80 2263 2266 12350003 10.2527/2002.8092263x

[B38] Powell JE Visscher PM Goddard ME Reconciling the analysis of IBD and IBS in complex trait studies Nat Rev Genet 2010 11 800 805 10.1038/nrg2865 20877324

